# Triggering Growth via Growth Initiation Factors in Nature: A Putative Mechanism for *in situ* Cultivation of Previously Uncultivated Microorganisms

**DOI:** 10.3389/fmicb.2021.537194

**Published:** 2021-05-04

**Authors:** Dawoon Jung, Koshi Machida, Yoichi Nakao, Tomonori Kindaichi, Akiyoshi Ohashi, Yoshiteru Aoi

**Affiliations:** ^1^Department of Molecular Biotechnology, Graduate School of Advanced Sciences of Matter, Hiroshima University, Higashihiroshima, Japan; ^2^Research Institute for Science and Engineering, Waseda University, Tokyo, Japan; ^3^Department of Chemistry and Biochemistry, Graduate School of Advanced Science and Engineering, Waseda University, Tokyo, Japan; ^4^Department of Civil and Environmental Engineering, Graduate School of Engineering, Hiroshima University, Higashihiroshima, Japan; ^5^Unit of Biotechnology, Graduate School of Integrated Sciences for Life, Hiroshima University, Higashihiroshima, Japan

**Keywords:** uncultured microbes, cultivation, resuscitation, non-growing, dormancy, diffusion chamber

## Abstract

Most microorganisms resist cultivation under standard laboratory conditions. On the other hand, cultivating microbes in a membrane-bound device incubated in nature (*in situ* cultivation) can be an effective approach to overcome this limitation. In the present study, we applied *in situ* cultivation to isolate diverse previously uncultivated marine sponge-associated microbes and comparatively analyzed this method’s efficiencies with those of the conventional method. Then, we attempted to investigate the key and previously unidentified mechanism of growing uncultivated microorganisms by *in situ* cultivation focusing on growth triggering via growth initiation factor. Significantly more novel and diverse microbial types were isolated via *in situ* cultivation than by standard direct plating (SDP). We hypothesized that some of environmental microorganisms which resist cultivation are in a non-growing state and require growth initiation factors for the recovery and that these can be provided from the environment (in this study from marine sponge). According to the hypothesis, the effect of the sponge extract on recovery on agar medium was compared between strains derived from *in situ* and SDP cultivation. Adding small amounts of the sponge extracts to the medium elevated the colony-formation efficiencies of the *in situ* strains at the starvation recovery step, while it showed no positive effect on that of SDP strains. Conversely, specific growth rates or saturated cell densities of all tested strains were not positively affected. These results indicate that, (1) the sponge extract contains chemical compounds that facilitate recovery of non-growing microbes, (2) these substances worked on the *in situ* strains, and (3) growth initiation factor in the sponge extract did not continuously promote growth activity but worked as triggers for regrowth (resuscitation from non-growing state).

## Introduction

Most microbes remain uncultivated and have been referred to as “mysterious dark matter” of the microbial world (Rinke et al., 2013; Lok, 2015). Cultivation independent surveys have demonstrated great diversity among these uncultivated species (Rappé and Giovannoni, 2003; Handelsman, 2004; Keller and Zengler, 2004). Accessing this “missing” microbial diversity is of great interest to both basic and applied sciences and has been regarded as a major challenge for microbiology.

Standard agar plating as a conventional method of cultivating microorganisms is limited because a significantly low proportion of the plated microbes readily form visible colonies on the agar plates, thus leading to plate count anomalies (Staley and Konopka, 1985; Amann et al., 1995). To overcome the limitations, much effort has been devoted to developing alternative approaches, including physically separating cells to decrease competition or inhibitors (Connon and Giovannoni, 2002; Zengler et al., 2002; Stingl et al., 2007; Tandogan et al., 2014), using modification to prepare agar media, using alternative gelling agents or antioxidants to minimize unfavorable compounds (Tamaki et al., 2005; Tanaka et al., 2014; Kato et al., 2018), and adding signal molecules or cocultivation with recruiter organisms to better reflect the natural environment (Bruns et al., 2002; Vartoukian et al., 2016; Tanaka et al., 2018). Nevertheless, most postulated extant microbes in nature remain uncultivated; thus, other essential factors for microbial cultivation that exist in nature are likely absent from those artificial conditions.

Previous studies have reported various types of microbial non-growing states such as viable but non-culturable (VBNC), near-zero growth (NZG) and dormancy (Lennon and Jones, 2011; Panikov et al., 2015; Pinto et al., 2015), which can be a potential cause for microbial uncultivability. Indeed, the “scout hypothesis” theory has proposed that such non-growing microbes wake up into active cells stochastically and the awakened cells can induce resuscitation of other dormant cells by secreting certain signaling molecules (Epstein, 2009; Buerger et al., 2012). A previous study proposed that cell-free extract from host microorganisms such as *Escherichia coli* was essential for the initiation of growth of specific bacterial strain, *Bdellovibrio bacteriovorus* (Ishiguro, 1973). However, no study has experimentally confirmed that the growth triggering of diverse environmental microbes from a non-growing state is a key phenomenon of microbial cultivation.

One simple solution for approaching the unknown growth factors is to incubate the microbes in their natural environment. Applying this idea to microbial cultivation led to developing *in situ* cultivation methods aiming to better simulate the natural environment (for a review, see Epstein et al., 2010). Several *in situ* cultivation methods with similar basic concepts have been applied to various environments, including sediment, activated sludge, alkaline soda lakes, sponges, soil, and a hot spring environment, and have been demonstrated to be highly capable of microbial cultivation (Bollmann et al., 2007; Aoi et al., 2009; Jung et al., 2013, 2014, 2016, 2018; Steinert et al., 2014). Recently developed new *in situ* cultivation method, diffusion bioreactor revealed that this approach is still useful for the cultivation of previously uncultured bacteria (Chaudhary et al., 2019). However, why these *in situ* cultivation techniques enable cultivating previously uncultivated microbes that are otherwise difficult or impossible to grow using conventional methods remains unclear. Answering this question would provide a key factor for microbial cultivation and contribute to developing additional advanced cultivation techniques.

In the present work, we applied *in situ* cultivation to a marine sponge, *Theonella swinhoei*, to isolate previously uncultivated microbial species. *Theonella swinhoei* as well as other sponge species is known as a repository of highly diverse bacterial community, mainly of the phyla *Actinobacteria*, *Chloroflexi*, *Cyanobacteria*, and *Proteobacteria* (Hentschel et al., 2002; Schmitt et al., 2012). Generally, marine sponges are rich sources of bioactive secondary metabolites of biotechnological interest for their antiviral, antitumoral, antimicrobial and cytotoxic properties (Koopmans et al., 2009; Mehbub et al., 2014; Anjum et al., 2016). It has been suggested that symbiotic microbes in marine sponges produces some of these bioactive metabolites (Brantley et al., 1995; Bewley et al., 1996; Flatt et al., 2005; König et al., 2006; Wang, 2006). Additionally, recently developed culture-independent approaches have provided molecular evidence of these microorganisms’ functional roles (Fan et al., 2012; Hentschel et al., 2012; Wilson et al., 2014). However, most of these producers remain unexplored and unavailable because the microbes associated with the sponges cannot be easily cultivated (Wang, 2006).

Extensive cultivation efforts have made use of several alternative techniques to isolate previously uncultured sponge-associated bacteria, including adding the sponge extracts or its skeleton to the media (Sipkema et al., 2011; Keren et al., 2016), using oligotrophic media (Schmidt et al., 2000), adding antibiotics to inhibit fast-growing bacteria (Selvin et al., 2009), using alternative gelling agent (Rygaard et al., 2017), and using diffusion devices (Jung et al., 2014; Steinert et al., 2014; Knobloch et al., 2019) and floating filters (Sipkema et al., 2011). These alternative approaches yielded an increased novelty of sponge isolates and improved cultivability rates up to 14% in some cases (Sipkema et al., 2011). However, most postulated extant microbes in sponges remain uncultivated; thus, further efforts to discover such novel microbes are needed.

One focus of the present study was to determine an effective cultivation method to broaden the accessible marine sponge-associated microbes. To do this, we employed an *in situ* cultivation method, the diffusion chamber, to isolate previously uncultivated marine sponge-associated microbes and comparatively analyzed this method’s efficiencies with those of the conventional method (direct agar plating). Although *in situ* cultivation devices have been applied to marine (Steinert et al., 2014) and freshwater sponges (Jung et al., 2014) in previous studies, the potential of this approach for cultivating novel, previously uncultivated microbial species from sponge samples has not well been clarified, and this approach has not been applied to the marine sponge *Theonella swinhoei.*

Another aim of the study is investigating the reason why *in situ* cultivation enables growing previously uncultivated microbes that cannot be isolated by conventional methods. We hypothesized that inactive or non-growing microbial cells are stimulated and aroused to begin regrowth during *in situ* incubation by a growth initiation factor from the outside environment and enriched inside an *in situ* cultivation chamber with nutrients. This brings different microbial groups, which mostly resist cultivation by conventional methods, to the culture collection. To test our hypothesis, recovery from the non-growing state in response to the marine sponge extract was comparatively analyzed between microbes isolated via *in situ* cultivation and those isolated via the conventional method.

## Results

### Identifying Isolates Based on the 16S rRNA Gene

**FIGURE 1:**
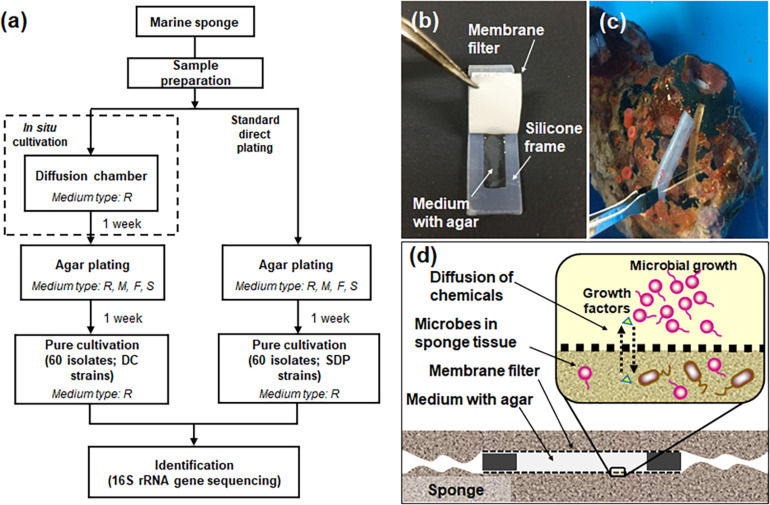
Flowchart of the experimental steps for microbial cultivation from marine sponges **(a)** and the diffusion chamber (DC) cultivation method used in the present study: a photo showing the structure of the chamber **(b)**, a photo showing installation of the DC device into the marine sponge **(c)**, a schematic image showing the principle of DC **(d)**. The letters R, M, F, and S in **(a)** refer to used media, 1/10 diluted R2A, Marine, Fish extract, and Sponge extract medium. See the experimental procedures for explanation of the different media used.

Figure 1 presents the overall experimental design for the cultivation experiments (Figure 1a), structure and application of the device, diffusion chamber (DC) (Figures 1b,C), and principle of *in situ* cultivation method (Figure 1d). Installation of the DC devices and 1 week incubation did not affect the health condition of sponges judged by the observation of its physical conditions. Indeed, it was alive after the experiment for more than 1 month.

**FIGURE 2:**
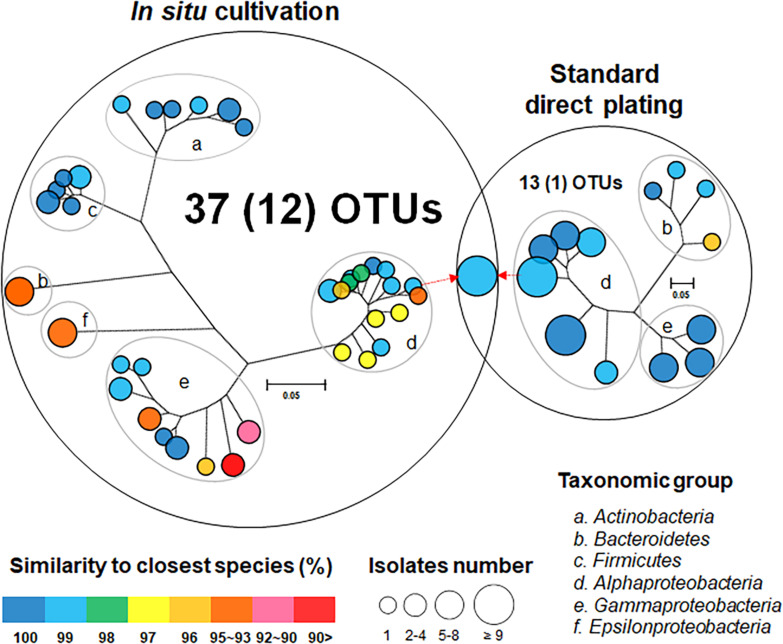
Venn diagram consisting of phylogenetic trees based on the 16S rRNA gene of isolates from each cultivation method. The trees are maximum-likelihood trees (fast bootstrap, 1,000 replicates). Circle size and color represent the number of OTUs (defined at 97% 16S rRNA gene sequence identity) and 16S rRNA similarity to the closest known relative in GenBank, respectively. Represented isolates are also grouped into taxonomic groups (a–f) at the phylum level (class level for *Alpha*, *Gamma*, and *Epsilon Proteobacteria*) by gray elliptical circles. Circles with pointed arrows indicate species that are overlapped among the isolates in the different cultivation methods. The numbers in parentheses show the numbers of novel species. The outer circle area corresponds to the number of isolated OTUs from each cultivation method.

One hundred twenty bacterial isolates (60 per cultivation method) were randomly selected and identified. *In situ* cultivation (DC) enabled isolating 37 species (defined as operational taxonomic units [OTUs] composed of 16S rRNA gene sequences sharing over 97% identity) from six taxonomic groups (*Actinobacteria, Bacteroidetes, Firmicutes, Alphaproteobacteria, Epsilonproteobacteria, and Gammaproteobacteria*) (Figure 2 and Supplementary Table 1). Standard direct plating (SDP) cultivation enabled isolating 13 species from three taxonomic groups (*Bacteroidetes, Alphaproteobacteria, and Gammaproteobacteria*) (Supplementary Table 2). Shannon-Weaver diversity indexes of isolated bacterial species from DC and SDP are 25.0, and 9.7, respectively. These results suggested that *in situ* cultivation (DC) yielded significantly higher diversity among the isolates at the species level than among those obtained via SDP cultivation (Figure 2). Only one species belonging to *Ruegeria atlantica* (99% similarity to the closest known species) was shared between the DC and SDP isolates, even though the inoculum used for each method was same.

**FIGURE 3:**
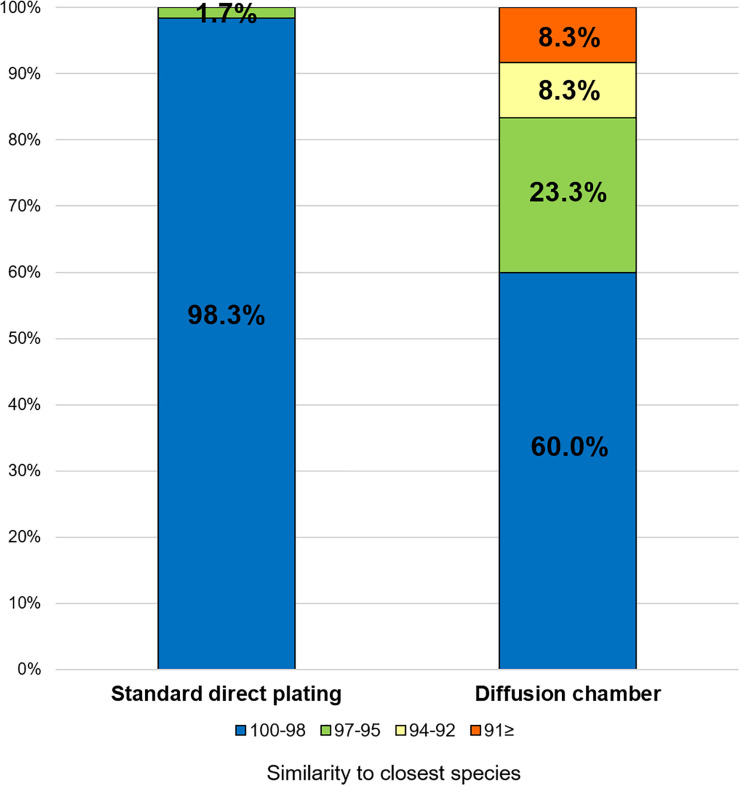
Similarity among the isolates from each cultivation method based on the 16S rRNA gene to the closest known relative in GenBank. Numbers in the bar graphs represent ratio (%) for each similarity level.

The ratios of novel species, defined as a strain with ≤ 97% 16S rRNA similarity to the closest known relative among the isolates, differed between the SDP and *in situ* cultivation methods. Only one SDP isolate (1.7%) was a novel species, while 40% (24 isolates belonging to 12 species) of the *in situ* isolates were novel species (Figures 2, 3).

We assigned all DC and SDP isolates to the closest OTUs, including uncultured strains, in GenBank and determined their environmental source. As a result, 42% of the closest OTUs of DC isolates originated from sponge or coral-associated samples, whereas only one (9%) of closest OTUs of SDP isolates were derived from such environments (Supplementary Tables 1, 2).

### Effect of the Sponge Extract on Cell Recovery on Agar Medium

To investigate the key mechanism of *in situ* cultivation which enabled isolation of novel bacteria by DC and not SDP, we picked 28 and 11 strains obtained from DC or SDP cultivation, respectively, which represented all identified OTUs, except some species which had failed to be sub-cultivated during further experiments (we hereafter define those selected stains as *in situ* and SDP strains, respectively).

**FIGURE 4:**
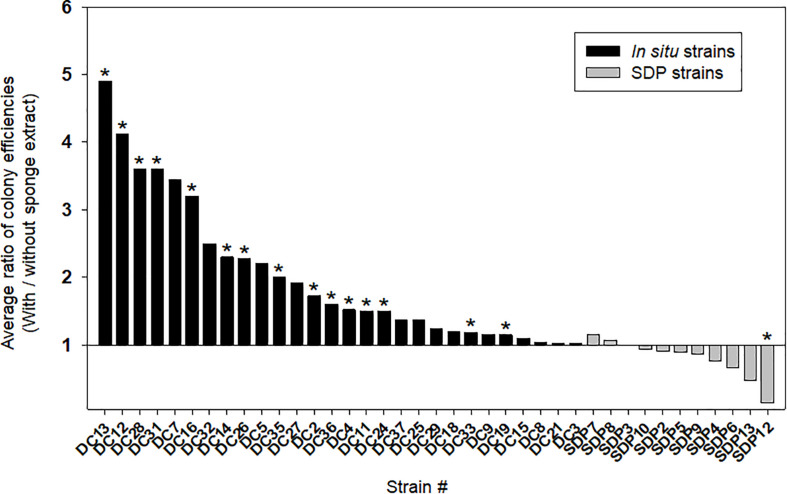
Effect of the sponge extract on starvation recovery of the *in situ* and SDP strains. The ratio of colony efficiencies between the value measured under two culture conditions (colony numbers on the agar medium with the sponge extract/colony numbers on the agar medium without the sponge extract) was calculated for each selected strain. The ratio was calculated with average value from triplicate measurements (*n* = 3) of the CFUs on each agar plate. The asterisks indicate that the value of bar graph is a significantly different between two conditions by statical analysis (*t*-test; *p* < 0.01).

Figure 4 shows the effect of adding the sponge extract (0.1% of the total volume) to the medium on starvation recovery (colony formation efficiency). Each strain’s colony efficiency ratio between the two culture conditions (with and without the sponge extract) was calculated (see Supplementary Figure 1 for absolute number of colonies). Adding the sponge extract to the medium exerted different effects on the recoveries between the *in situ* and SDP strains (Mann-Whitney *U*-Test; *P* < 0.05; Supplementary Figure 2A). The sponge extract positively affected the recoveries of most tested *in situ* strains. Among them, 15 strains (15/28, 54%) showed significantly different colony numbers on agar media between two conditions, with and without sponge extract (Figure 4 and Supplementary Figure 1). In contrast, none of SDP strains was positively affected except one SDP strain negatively affected by the sponge extract addition (Figure 4). Note that one of the SDP strain which were slightly positive affected by addition of sponge extract is *R. atlantica*. This species was also identified in *in situ* isolates (Supplementary Tables 1, 2).

### Effect of the Sponge Extract on Specific Growth Rates and Saturated Cell Densities

**FIGURE 5:**
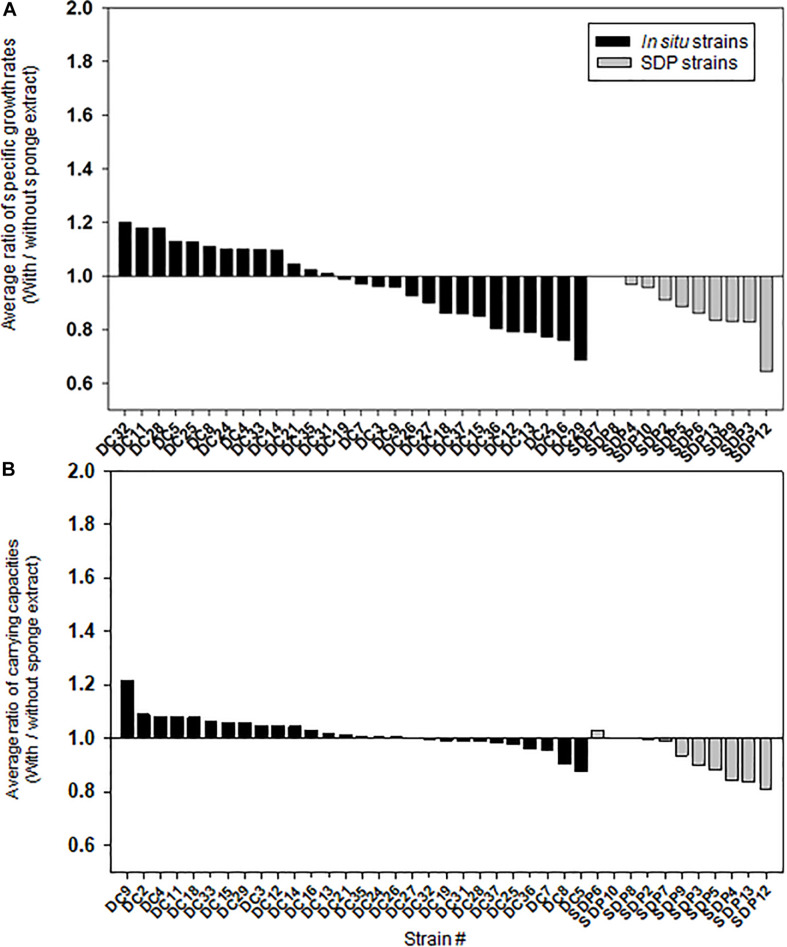
Effect of the sponge extract on specific growth rate **(A)** and saturated cell density **(B)** of the *in situ* and SDP strains. The ratio of the specific growth rate (μ) between the value measured under the two culture conditions (μ with the sponge extract/μ without the sponge extract) was calculated for each tested strain **(A)**. The ratio of the saturated cell density (maximum value of OD_600_) between the value measured under the two culture conditions (saturated cell density with the sponge extract/saturated cell density without the sponge extract) was calculated for each tested strain **(B)**. The ratio of specific growth rate and saturated cell density was calculated with average value from triplicate experiments (*n* = 3).

Figures 5A,B shows the effect of adding the sponge extract (0.1% of the total volume) to the medium on the specific growth rate and saturated cell density (carrying capacities), respectively. Each strain’s specific growth rate and saturated cell density were measured and compared under two medium conditions (with and without the sponge extract; See Supplementary Figure 3 for growth curves of selected strains) to examine the sponge extract’s effect on growth promoting (Supplementary Figures 4, 5). In contrast to its effect on starvation recovery (colony forming efficiencies) of *in situ* strains, adding the sponge extract to the medium neither positively affected the specific growth rate nor the saturated cell density of both strain types (Figure 5 and Supplementary Figure 2), while saturated cell densities of SDP strains were more negatively affected compared with that of *in situ* strains (Mann-Whitney *U*-Test; *P* < 0.05; Supplementary Figures 2B,C). The addition slightly negatively affected the specific growth rates for more than half the tested *in situ* strains (15/28, 54%; Figure 5A) and all SDP strains except SDP7 and SDP8 (SDP8 corresponds to *R*. *atlantica*, also isolated via *in situ* conditions).

## Discussion

We demonstrated that significantly more novel (previously uncultivated types) and diverse microbial species were isolated via *in situ* cultivation than by standard direct plating (SDP). Then, we observed that (1) the sponge extract contains chemical compounds (potential growth initiation factor) that facilitate recovery of some bacteria, and (2) addition of the sponge extract to the medium did not continuously promote growth activity but worked as triggers for regrowth (resuscitation from dormancy). To our knowledge, the present study shows the first observation that the potential growth initiation factor (signaling-like compounds) in natural environments stimulate microbial resuscitation from a non-growing state, suggesting that triggering growth might be a key for cultivating previously uncultivated microbes.

### Effectiveness of *in situ* Cultivation Techniques for Isolating Novel Species From Marine Sponges

Marine sponges are sources of many bioactive natural products (Schmidt et al., 2000; Piel et al., 2004; Wilson et al., 2014), which are often produced by host-specific microbes that are mostly unknown because of their uncultivability (Taylor et al., 2007; Fan et al., 2012). Molecular surveys have shown that sponges host rich microbial communities (Schirmer et al., 2005; Thomas et al., 2016); however, only a minor component of this richness has been cultivated via the conventional cultivation method (Wang, 2006).

Here, we isolated previously uncultivated microorganisms from the marine sponge *Theonella swinhoei*. In total, 40% of the *in situ* isolates were uncharacterized, which is a higher percentage than previously reported in similar studies, albeit with other sponge species (Jung et al., 2014; Steinert et al., 2014). Newly discovered microbes are candidates as sources for valuable secondary metabolites, and the newly established approach would be a strong tool for further accessing untapped microbial resources.

### A Key Mechanism of *in situ* Cultivation for Growing Previously Uncultivated Microbial Types

*In situ* cultivation approach via diffusion chamber (DC) enabled obtaining a different culture collection that was larger and more novel than that obtained by standard direct plating (SDP) (Figures 2, 3). Only one species was common in culture collections between the *in situ* and SDP cultivation methods. These results indicated that most isolated species were unique to their isolation approach, and *in situ* cultivation enabled cultivating specific microbial types that have rarely been isolated by conventional cultivation approaches. These results raised the question, “what mechanism from the alternative approach yielded these previously uncultivated microbial groups that differed entirely from the standard cultivation approach?” A potential explanation is that during *in situ* cultivation, inoculated microbes receive unknown growth components from the natural environment necessary for their growth but absent from the artificial medium (Kaeberlein et al., 2002). However, the present study found that isolates unique to *in situ* cultivation grew stably under the same conditions as those of the SDP cultivation at the sub cultivation step, which lacks such growth components. This phenomenon also occurred in previous studies that used *in situ* cultivation (Bollmann et al., 2007; Jung et al., 2013, 2014, 2018). Only the difference between the *in situ* and SDP cultivation methods in the experimental procedure was whether a 1 week incubation was performed in the natural environment (inside the marine sponge in this study) prior to agar plate cultivation (Figure 1a). We then explored what mechanism could explain this observation. We assumed that some factor presents in nature induced non-growing microbes to initiate growth and this factor should be also included in the sponge extract.

### Growth Triggering by Growth Initiation Factor in Nature

To investigate the mechanism behind the *in situ* cultivation results, the effect of adding small amounts of the sponge extract on the cell recoveries on agar medium (colony forming efficiencies) and specific growth rates and saturated cell density of representative isolates (from every species) were compared between the strains derived from *in situ* and SDP cultivation.

Consequently, adding the sponge extract to the medium (0.1% of the total volume) elevated the colony formation efficiencies of all tested *in situ* strains at the starvation recovery step, while it only negatively affected that of the SDP strains (Figure 4). This indicates that the sponge extract contains a factor facilitating recovery (likely to be growth initiation factor) and these substances perhaps selectively work on *in situ* strains. In contrast, adding the sponge extract did not elevate the specific growth rates, nor carrying capacities (saturated cell densities) in either the *in situ* or SDP strains but rather slightly suppressed them (Figures 5A,B). This indicates that elevating colony formation efficiencies by adding the sponge extract was not due to supplying factors simply promoting growth activity nor by supplying nutrient for cell growth. These observations suggest that the sponge extract contains a growth initiation factor (signaling-like compounds), which does not continuously promote growth activity but works as trigger for regrowth, which is likely resuscitation from dormancy (non-growing state).

**FIGURE 6:**
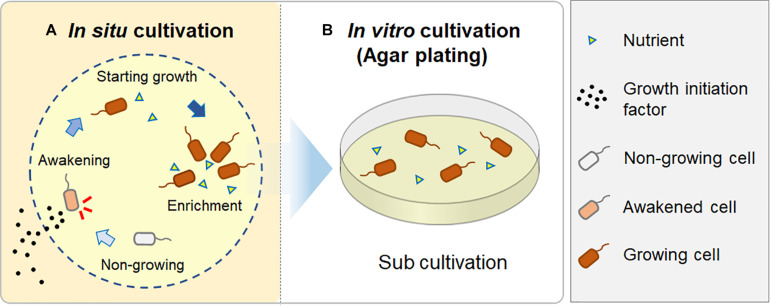
A hypothesis for how *in situ* cultivation yields different isolates from microbial recovery in a natural environment. During *in situ* cultivation, dormant microbes are induced from a non-growing state by growth initiation factor from the outside environment. They then start to grow and become enriched, supported by sufficient nutrients in the chamber **(A)**; when they are sub-cultured after the *in situ* cultivation, they continuously grow on an agar plate, resulting in visible colony appearance **(B)**.

Therefore, we hypothesized that during the *in situ* cultivation, such signaling-like compounds, potential growth initiation factor, have been provided from the natural environment (i.e., the marine sponge), while nutrients were provided inside the chamber from the beginning. These factors would enable microbes to resume growth and finally become enriched inside the chamber, because the nutrients supplied in the chamber could support their continuous growth (Figure 6). In contrast, standard agar plate medium lacks such growth initiation factor; thus, it is likely that significant numbers of inoculated microbial cells did not resuscitate from non-growing state and thus did not form colonies.

Because SDP strains require no growth initiation factor to begin regrowing, these strains are frequently isolated via conventional approaches (the SDP method in the present study) and have been discovered in previous studies. Therefore, microbial types in isolates differ completely between methods, and the novelty and diversity of isolates derived from *in situ* cultivation are higher than are those derived from the conventional approach.

For cell recovery and growth promotion tests, sponge extract was added to the medium first followed by autoclaving together, due to the convenience of experiments. We have tested both autoclaved and unautoclaved versions as a pretest and found out unautoclaved (sterilized by filtration) sponge extract as well as autoclaved possessed the activity letting the microbes recover from starvation (growth initiation). These suggest that autoclaved sponge extract remains key function and worked for our purpose, and the growth initiation factor is likely to be a thermostable compound. In previous studies, autoclaved sponge extracts were also used and proved its efficiency for the growth media (Selvin et al., 2009; Kaboré et al., 2019) and the experiments to figure out interaction between sponge extract and bacterial growths (Esteves et al., 2017) suggesting various thermostable compounds generally contains in sponge extract.

### Significance of the Present Study Relative to Solving Microbial Uncultivability

Various *in situ* cultivation methods with similar basic rationales have been applied to diverse environmental samples and have been shown to be highly capable of microbial cultivation compared with conventional approaches because the method led to isolating some uncultivated and phylogenetically new and industrially important isolates (Sizova et al., 2012; Ling et al., 2015; Berdy et al., 2017). However, no study has clarified the growth facilitation mechanism of *in situ* cultivation or the reason why *in situ* cultivation yields more phylogenetically novel and diverse microbial types than does the conventional method. This has limited further advancing the method regardless of its potential. Therefore, the present study clarifying the growth facilitation mechanism is the first step toward the resolving this issue.

Furthermore, the discovery in the present study, especially regarding microbial resuscitation from a non-growing state in response to the growth initiation factor gives us an important clue toward solving the challenging issue of why most environmental microbes cannot grow on standard agar media. Several studies have been conducted to determine the reasons for microbial uncultivability, such as inoculum size (Davis et al., 2005) and phosphate-catalyzed hydrogen peroxide formation from agar (Kawasaki and Kamagata, 2017). A few attempts have been made to discover microbial interactions via signaling molecules that promote the growth of specific microbial types. Short peptides (Nichols et al., 2008), siderophores (D’Onofrio et al., 2010), and quinones (Fenn et al., 2017) have been identified as growth-promoting factors for microbial types that cannot grow independently.

However, the causes of the uncultivability and the mechanism facilitating growth of previously uncultivated microbes shown in the present study differ from those reported in previous studies in several aspects as follows. Firstly, no study has focused on growth triggering. In other words, there is no study distinguishing “simple growth promotion” from “growth triggering.” However, this would be one of key to solving the microbial uncultivability as shown here. Second, the “scout hypothesis” theory has been proposed so far, which states that (1) dormant microbes stochastically and spontaneously awaken, and (2) awakened cells trigger the awakening of other dormant cells (Epstein, 2009; Buerger et al., 2012). However, no study has provided experimental evidence that growth triggering of environmental microbes from a non-growing state is a key phenomenon of microbial cultivation.

Although *in situ* cultivation method used in this study demonstrated to be highly capable of isolation of novel species, there are still numerous uncultured species that were represented in culture-independent methods (Schmidt et al., 2000; Sipkema et al., 2011; Hentschel et al., 2012; Thomas et al., 2016) but not in our culture collection. In this study, we focused on microbial types that requires “factors” provided from nature to initiate growth. However, there must be several other important factors to cultivate uncultivated microbial types. Therefore, application of other methods or development of new cultivation techniques need to be taken into consideration to enlarge the microbial diversity in culture collection. In addition to effort on designing new medium types, targeting extremely slow growing microbes, and microbial types which cannot grow above certain cell density (lower than detection limit) should be effective for cultivation of uncultivated.

Next challenge beyond the present study include (1) identifying the growth initiation factor via natural chemical approaches and (2) clarifying the donors and accepters of signaling compounds (i.e., who produces what and to whom). This will provide a new insight into microorganisms previously considered uncultivable and the complex networking system for controlling growth among microbes in nature.

## Materials and Methods

### Sample Collection

Marine sponge (*Theonella swinhoei*) specimens were collected while scuba diving at 15–20 m depth in August 2015, near Okino island, Kochi prefecture, Japan. Sponges were kept in a cooling box with seawater and transported to the aquarium and laboratory for further experiments.

### Media

We used the following media for agar plating (for both standard direct plating method and sub cultivation for *in situ* cultivation; Figure 1a). Four types of non-selective media were used in this study in order to cover diverse microbial species, which were adapted from previous studies isolating wide variety of sponge-associated bacteria (Subbannayya et al., 2002; Sipkema et al., 2011; Jung et al., 2014): (1) 1:10 diluted Reasoner’s 2A (R2A) media (10% of the manufacturer’s suggested concentration, Nihon Seiyaku, Japan); (2) marine media (Difco, Franklin Lakes, NJ, United States); (3) fish extract media (0.2 g fish extract and 0.1 g yeast extract per liter); and (4) sponge extract media (0.01 g peptone and 40 ml aqueous sponge extract per liter). The sponge extract was prepared by mixing homogenized sponge and sterile distilled water including 3.5% artificial sea salt at a 1:1 (vol/vol) ratio, then vortexing for 1 min, spinning down (10 min, 8,000 rpm), and filter sterilization using a 0.2-μm pore-size filter. All media contained 1.5% agar to produce solid medium, and all media except the marine media were supplemented with 3.5% artificial sea salt, SEALIFE (Marine Tech, Tokyo, Japan). Colonies grown on these media were subcultured for purification on 1.5% agar plates supplemented with 1:10 R2A broth. All isolates grew well on 1:10 diluted R2A agar media.

### Diffusion Chamber *in situ* Cultivation

Diffusion chamber (DC) *in situ* cultivation method was performed to isolate marine-sponge associated microbes. The DC was prepared as described previously (Bollmann et al., 2007). The DC is an *in situ* cultivation device that allows exchanging of chemical compounds, thereby simulating a natural environment (Figure 1d). To construct a chamber, a 0.1 μm pore-size filter polycarbonate membrane (Millipore, Darmstadt, Germany) was glued to a rectangular-shaped silicone rubber frame with a waterproof adhesive (Cemedain, Tokyo, Japan) (Figure 1b). The frame was made of silicone rubber sheet (*w* = 3 cm, *l* = 1.2 cm, *d* = 0.3 cm), and cut to an internal size of 2 cm by 0.44 cm. To prepare the marine sponge sample inoculum, 20 g of the subsample was rinsed three times with sterile artificial sea water and pulverized using a sterile mortar and pestle with the same volume of sterile artificial sea water. The aliquots were diluted 10^–4^–10^–5^ with sterilized water including 3.5% artificial sea salt and mixed with warm (45°C) agar with 1:10 diluted R2A. The mix was placed on the chamber membrane, and the second membrane was glued to the other side of the silicone flame, sealing the agar inside to form a chamber.

For *in situ* cultivation, the sponges were kept in aquariums in the Takehara station of Hiroshima University in Takehara-city, Japan. The aquarium was a cylindrical container with a holding capacity of ca. 43.3 L (Ø = 0.35 m, *d* = 0.45 m), and seawater was supplied continuously. The temperature of seawater in aquarium was controlled at 15–20°C by using aquarium heater (Gex, Osaka, Japan). To install the DC into the sponge, similar sized grooves as those of the device were made on the specimen’s surface by cutting with a scalpel blade, and four DCs were inserted to two sponges (Figure 1c). After 1 week of *in situ* incubation, DCs were retrieved from the sponges using tweezers and then transported to the laboratory. Physical conditions of the sponges such as size, color, hardness and smell were observed after cultivation to check its health conditions that may affect to community of symbiont. The surface of DC was washed using sterilized water including 3.5% artificial sea salt and dried. Then the device was opened, and the agar with the grown material was carefully taken out of the chamber. The agar material from each device was added to 1 ml of sterilized water including 3.5% artificial sea salt, homogenized with a sterile stick, vortexed, diluted in a 10-fold series from 10^–1^ to 10^–4^ using sterile water including 3.5% artificial sea salt, subcultured on media as described above with three replicates per each dilution, and incubated at 20°C. After 1 week, 60 colonies were randomly selected, pure cultured using 1:10 R2A medium and used for further analysis.

### Standard Direct Plating Cultivation

Conventional standard direct plating (SDP) was performed to compare the SDP results with those of the *in situ* cultivation approaches (Figure 1a). The same inoculum used for DC cultivation was diluted serially and plated directly onto the agar media as described above, with three replicates. After incubating for 1 week at 20°C, 60 colonies were randomly selected and pure-cultured using 1:10 R2A medium for microbial identification.

### Identification of Isolates Based on 16S rRNA Gene Sequencing

Taxonomic identification was performed by sequencing based on 610- to 712-bp long fragments of the 16S rRNA gene. The colony material was used directly as a PCR template. The 16S rRNA gene was amplified using the universal primers, 27F (5′-AGAGTTTGATCCTGGCTCAG-3′) and 1492R (5′-GGTTACCTTGTTACGACTT-3′), with a KOD FX Neo system (Toyobo, Osaka, Japan), then purified using a fast gene purification kit (Nippon Genetics, Tokyo, Japan). The purified PCR products were sequenced with a commercial sequencer (Takara Bio, Shiga, Japan) by fluorescent dye terminator sequencing. The sequences were compared with those available in GenBank^1^ using Molecular Evolutionary Genetics Analysis (MEGA software, Tempe, AZ, United States) to determine their closest relatives. Distance matrices and phylogenetic trees based on 16S rRNA sequences were calculated among the isolated OTUs (defined at 97% 16S rRNA gene sequence identity) according to the Kimura two-parameter model and neighbor-joining algorithms using the MEGA program (MEGA software, Tempe, AZ, United States). Newly generated sequence data has been deposited in GenBank (see footnote) under accession numbers MK672856 to MK672868 for isolates from the DC, and MK674856 to MK674892 for isolates from SDP cultivation.

### Effect of the Sponge Extract on Starvation Recovery

The colony formation efficiency ratios between the two culture conditions (media with and without the sponge extract) were calculated for each tested strain to examine the effect of the sponge extract on starvation recovery. For the test, we used 28 and 11 strains obtained from *in situ* and SDP cultivation (out of 60 isolates each), respectively, which represented all identified OTUs except for a few species (9 and 2 species out of 37 DC and 13 SDP species, respectively, had been lost their growth activity during further experiments). First, selected strains were cultured in 5 ml of 1:10 diluted R2A broth with 3.5% artificial sea salt at 20°C (liquid cultures) with shaking at 150 rpm for 3–5 days (strains were harvested at the beginning of stationary phase). To detect the beginning of the stationary phase, the optical density (OD) was measured at 600 nm using a spectrophotometer (DR 3900, HACH, Metropolitan, MD, United States). Strains were then diluted with artificial sea water (1:100) followed by incubation at 5°C for 3 days to inactivate them (Oliver, 2005; Lennon and Jones, 2011). As test strains after the pre-cultivation were diluted in 100-fold with artificial seawater, this process made microbial cells starved under low temperature conditions. After 3 days, the liquid culture serial dilutions were inoculated in triplicate on two types of agar media: 1:10-diluted R2A agar medium with 0.1% (vol/vol) of the sponge extract and the same medium without the sponge extract. The sponge extract was prepared by mixing homogenized sponge and sterile distilled water, including 3.5% artificial sea salt at a 1:1 (vol/vol) ratio, then vortexing for 1 min, centrifuging (10 min, 8,000 rpm), and filter sterilization using a 0.3 μm pore-size filter. The sponge extract was added to the medium before autoclaving. After 5 days of incubation at 20°C, the CFUs on each agar plate were counted. Dilutions that resulted in 30–500 CFUs were selected for the colony counting. The colony number ratios between the two culture conditions (with and without the sponge extract) were measured for each tested strain. The colony numbers of each strain were also compared between the two conditions using the *t*-test. The significance level was set to *P* < 0.01. Uninoculated agar plates per each condition were used as negative control.

### Effect of the Sponge Extract on the Specific Growth Rate and Saturated Cell Density

Specific growth rates and saturated cell densities under the two culture conditions (medium with and without the sponge extract) were measured and compared for each tested strain to examine the sponge extract’s effect on the growth activity. First, one strain per each OTU from the DC and SDP cultivations (same strains employed for testing starvation recovery) were cultured in the same manner as described above. Next, 5–20 μl of the microbial cell suspension from each liquid culture was inoculated into the two media (1:10-diluted R2A medium with 0.1% [vol/vol] of the sponge extract and the same medium without the sponge extract) in triplicate. The cultures were then incubated at 20°C with shaking at 150 rpm. The optical density (OD) was measured at 600 nm using a spectrophotometer (DR 3900, HACH, Metropolitan, MD, United States). The values of OD 600 were measured at 2 h (or 3 h in few cases that some strains grew for a long time) intervals and until the growth curves reached stationary phase. The growth curve for the OD 600 value was fitted using the non-linear regression function with a logistic model in Sigma plot software (San Jose, CA, United States, Systat Software, Inc.). Specific growth rates, as maximum rates of change, were determined to μ by the change in OD_600_ on a logarithmic scale as time. The ratio of the specific growth rate (μ) between the values measured under the two culture conditions (with and without the sponge extract) was calculated for each tested strain. The saturated cell density (maximum growth) was determined by its maximum value on the fitted growth curve. The ratio of colony efficiencies, specific growth rates, carrying capacities (with/without sponge extract) was compared among cultivation methods using the Mann-Whitney *U*-Test. The significance level was set to *P* < 0.05.

## Data Availability Statement

The datasets generated for this study can be found in the GenBank: MK672856 to MK672868 and MK674856 to MK674892.

## Ethics Statement

Ethical review and approval was not required for the animal study because marine sponge (the animal used in this study) is exempt from the regulation on animal research.

## Author Contributions

DJ, YN, and YA conceived and designed the study. DJ and KM performed the laboratory experiments. DJ, TK, AO, and YA analyzed the data and compiled the results. DJ and YA wrote the manuscript draft. All authors revised the draft, approved the final manuscript version and were accountable for all aspect of the work.

## Conflict of Interest

The authors declare that the research was conducted in the absence of any commercial or financial relationships that could be construed as a potential conflict of interest.

## Footnotes

1 www.ncbi.nlm.nih.gov
