# Supplementary material for: Triggering Growth via Growth Initiation Factors in Nature: A Putative Mechanism for in situ Cultivation of Previously Uncultivated Microorganisms
Source: Front Microbiol. 2021 May 4;12:537194. doi: 10.3389/fmicb.2021.537194 (PMC8129545; doi:10.3389/fmicb.2021.537194)
Supplement: Supplementary file 1 [file Data_Sheet_1.pdf]

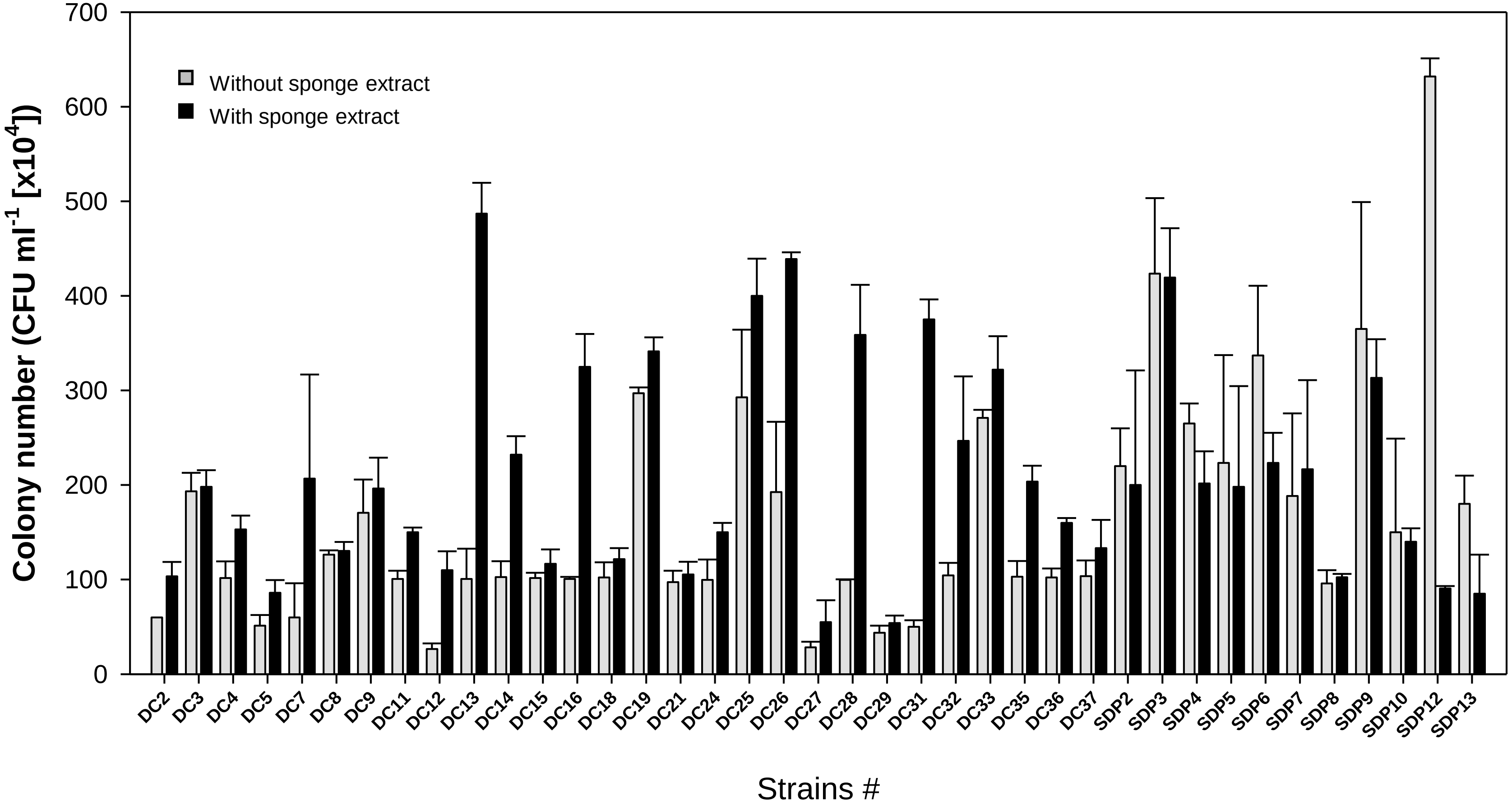

Fig. S1. Effect of the sponge extract on starvation recovery of the *in situ* and SDP strains. Each selected *in situ* and SDP strain was inoculated in triplicate on two types of agar media: 1:10-diluted R2A agar medium with 0.1% (vol/vol) of the sponge extract and the same medium without the sponge extract. The error bars represent standard deviation of the mean value.

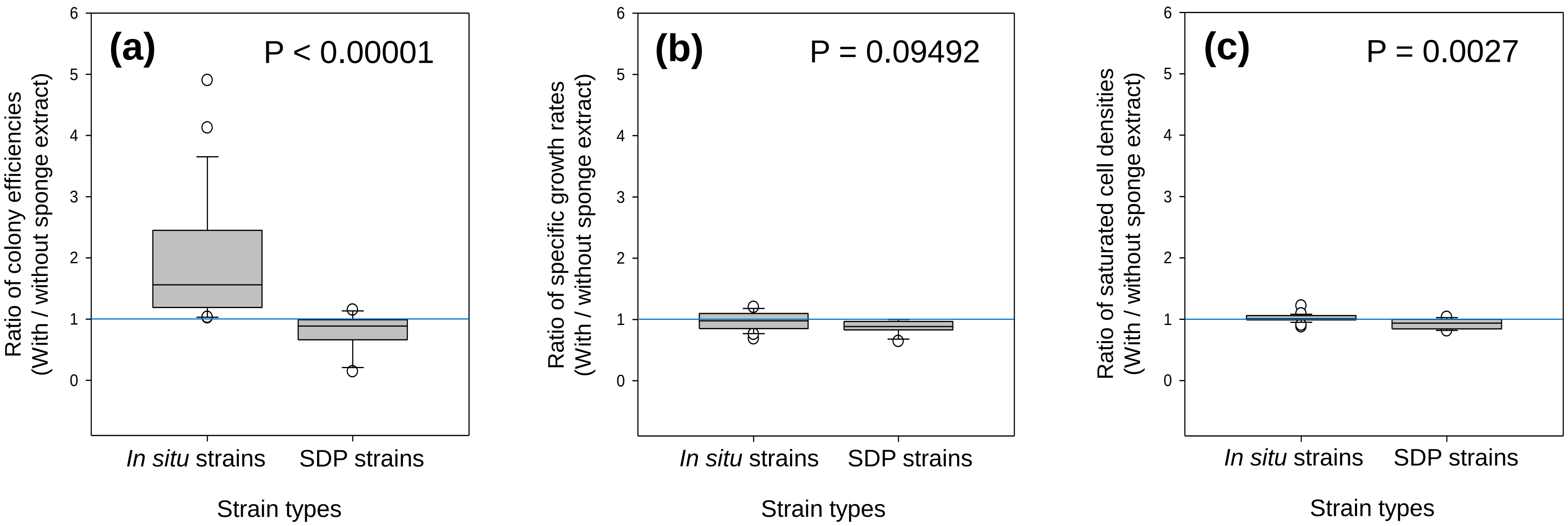

Fig. S2. Box-and-whisker plots of effect of the sponge extract on starvation recovery (a), growth rate (b) and saturated cell density (c) of *in situ* and SDP strains. The line inside each box indicate the median value. Lines extending from the boxes represent minimum and maximum values. The blue line indicates the ratio of 1.0. The ratio of colony efficiencies, specific growth rates, saturated cell densities (with/without sponge extract) was compared among strain types using the Mann-Whitney U Test procedure.

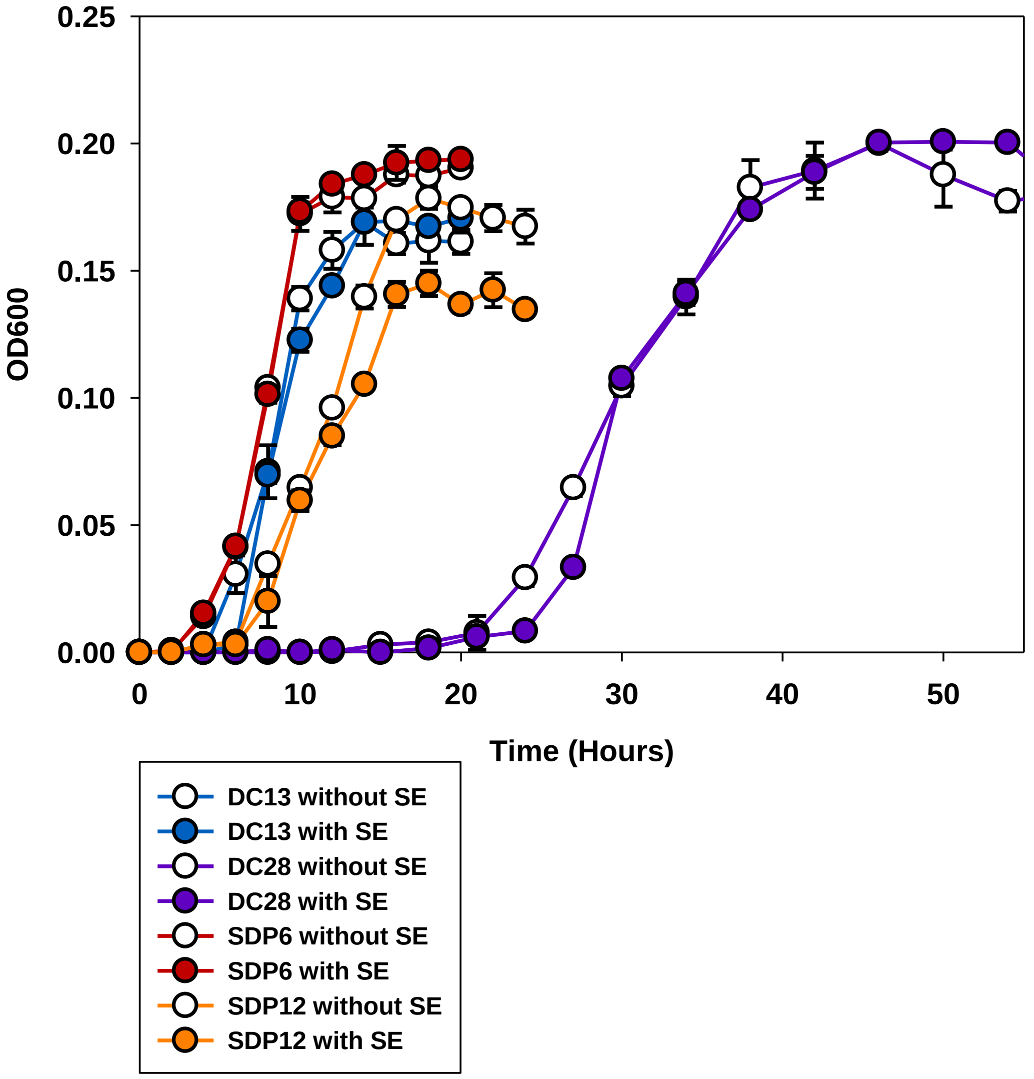

Fig. S3. The growth curves of four representatives to show how the experiment have been performed to measure the specific growth rate and saturated cell density.

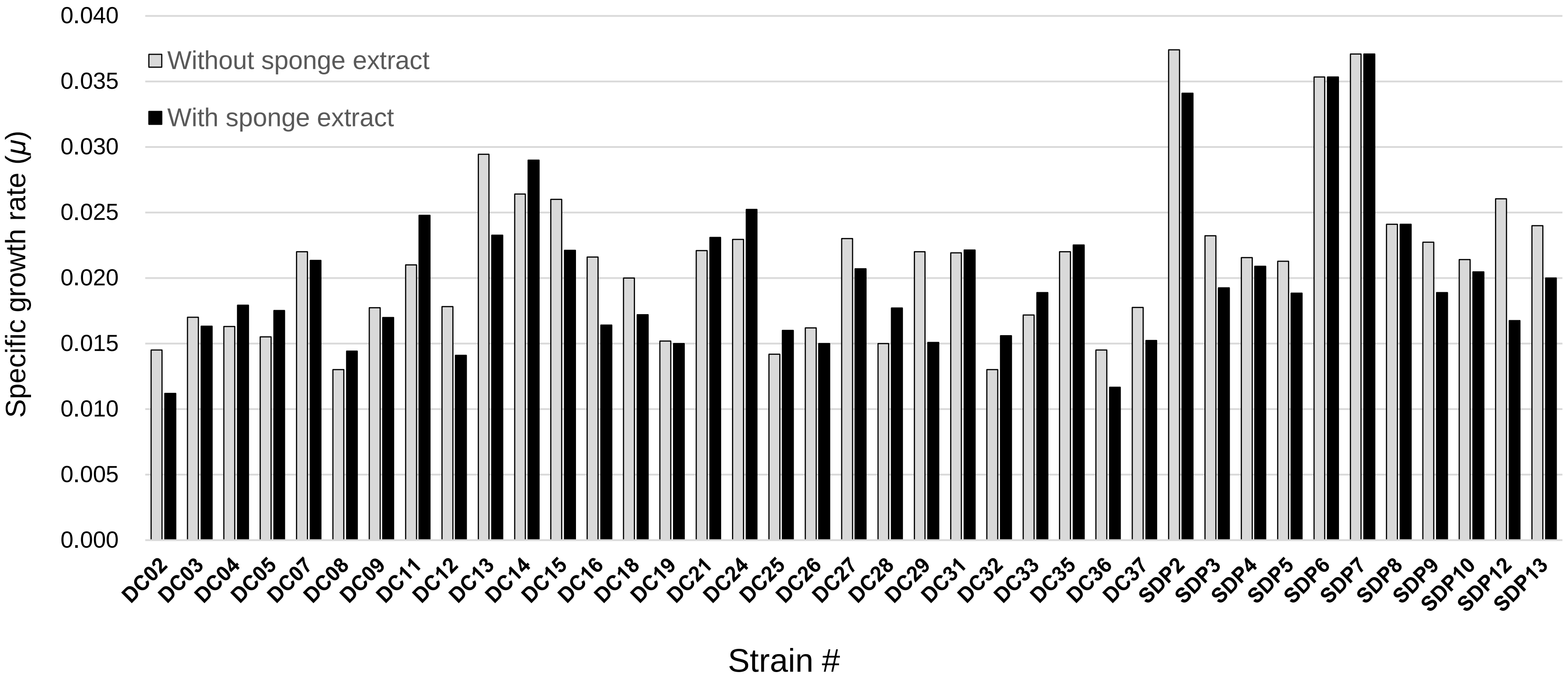

Fig. S4. The specific growth rate for each tested strain measured under the two culture conditions (the medium with and without the sponge extract).

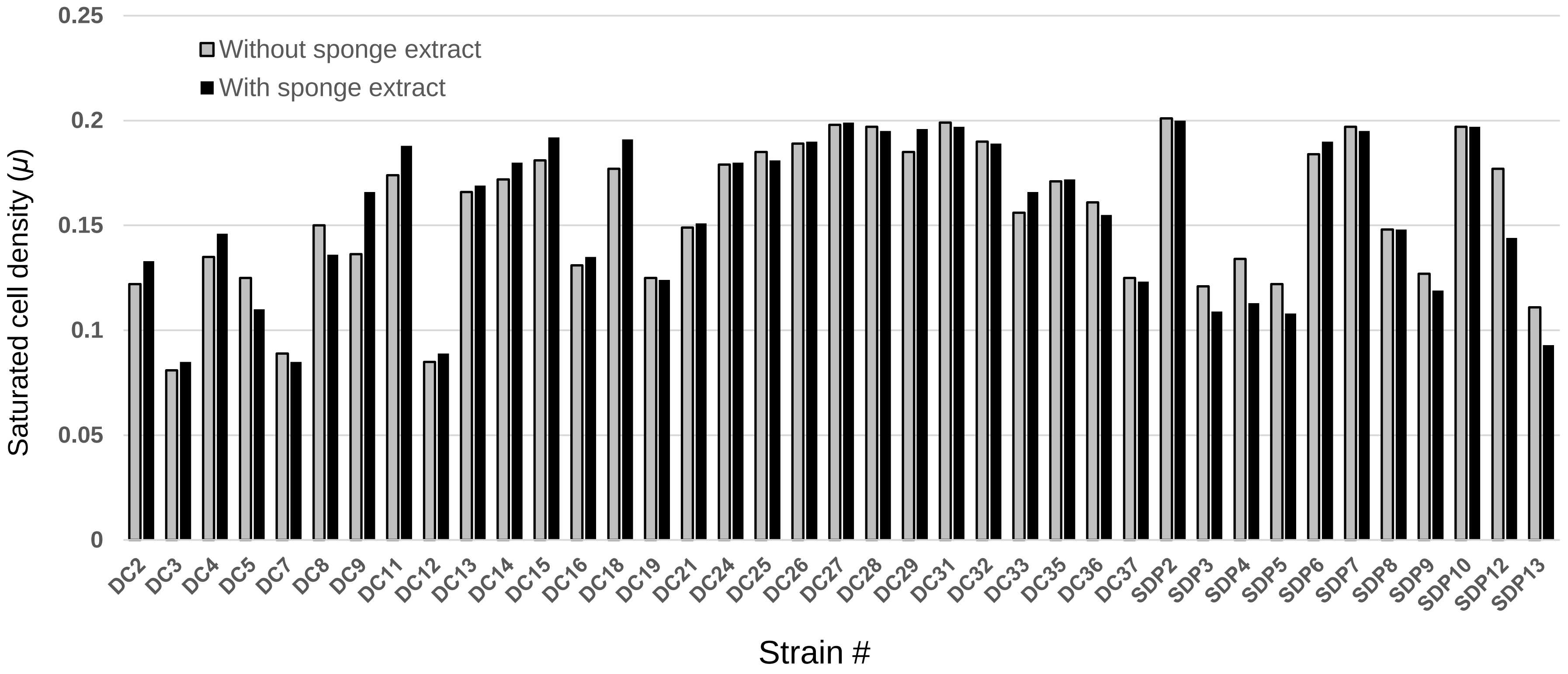

Fig. S5. The saturated cell density for each tested strain measured under the two culture conditions (the medium with and without the sponge extract).
