# Supplementary material for: Triggering Growth via Growth Initiation Factors in Nature: A Putative Mechanism for in situ Cultivation of Previously Uncultivated Microorganisms
Source: Front Microbiol. 2021 May 4;12:537194. doi: 10.3389/fmicb.2021.537194 (PMC8129545; doi:10.3389/fmicb.2021.537194)
Supplement: Supplementary file 2 [file Data_Sheet_2.PDF]

Table S1. Phylogenetic affiliations of isolates with the DC method on the basis of 16S rRNA gene sequences.

Taxonomic group	Strain #	Closeast species among cultured stains	Similarity	Number of isolates	Medium[a]	Closest sequences among uncultured / environmental sample[b]	Environmental source	Accession # of reference
Actinobacteria	DC1	Streptomyces angustmyceticus	100	1	F	Uncultured Streptomyces sp. clone CP-5	soil	KM213013
	DC2	Streptomyces clavuligerus	99	2	R	Uncultured actinobacterium clone FI-1M_H01	soil	EF220632
	DC3	Streptomyces galbus	100	1	M	Uncultured bacterium clone BFO1050	soil	KT460542
	DC4	Streptomyces pulveraceus	100	1	S	Uncultured Streptomyces sp. clone VII22-5-3	soil	KC898841
	DC5	Streptomyces sanglieri	100	2	F, R	Uncultured actinobacterium clone SL6a/8	forest soil	EU715914
Bacteroidetes	DC6	Marinifilum flexuosum	94	5	F, M, R	Uncultured bacterium clone Woods-Hole_a3803	Ciona intestinalis	KF799405
Firmicutes	DC7	Bacillus cereus	100	2	M, R	Unidentified microorganism clone oclvp30r2a200100	coastal water	MG271390
	DC8	Bacillus hunanensis	100	1	M	Uncultured Bacillus sp. clone TGRWLFZ-16s-SI307	inundated soil	KT122287
	DC9	Bacillus lehensis	100	1	R	Uncultured Bacillaceae bacterium clone: m1	termite gut	AB111971
	DC10	Bacillus thermophilus	99	1	F	Uncultured Bacillus sp. clone TGRWLFZ	inundated soil	KT122287
	DC11	Lysinibacillus sphaericus	100	1	S	Uncultured bacterium clone NT183	termite	KR779446
	DC12	Paenibacillus harenae	98	1	S	Uncultured Paenibacillus sp. clone B4	decomposition	HQ154650
Alphaproteobacteria	DC13	Leisingera aquimarina	98	1	S	Uncultured bacterium clone en1385-93	marine sponge	JQ240729
	DC14	Pelagicola litoralis	98	2	R, S	Uncultured bacterium clone Woods-Hole_a3989	Ciona intestinalis	KF799329
	DC15	Pelagimonas varians	99	3	F, M	Uncultured bacterium gene clone: 3MP-B-2AC-58	methane process	AB731294
	DC16	Phaeobacter arcticus	99	1	R	Uncultured bacterium clone 135-76	marine sediment	MF978509
	DC17	Phaeobacter daeponensis	99	1	F	Uncultured bacterium clone SHFG707	coral tissue	FJ203288
	DC18	Phaeobacter gallaeciensis	97	1	M	No reference	-	-
	DC19	Roseovarius aestuarii	99	1	M	Uncultured bacterium clone TD9G11	coral	GQ301415
	DC20	Roseovarius sediminilitoris	99	1	M	Uncultured bacterium clone SanDiego_a6421	Ciona intestinalis	KF799735
	DC21	Ruegeria atlantica	99	2	R, S	Uncultured alpha proteobacterium clone 3.1	sea urchin	KT367734
	DC22	Ruegeria conchae	99	1	F	Uncultured bacterium clone QAMU9	coral tissue	KU354042
	DC23	Shimia marina	97	1	R	No reference	-	-
	DC24	Sulfitobacter pacificus	100	1	M	Uncultured bacterium clone Woods-Hole_a1731	Ciona intestinalis	KF798502
	DC25	Thalassobius aestuarii	97	1	M	Uncultured bacterium clone ep1385-09	marine sponge	JQ240828
	DC26	Tropicibacter phthalicicus	96	1	M	No reference	-	-
	DC27	Vadicella arenosi	97	1	M	No reference	-	-
Epsilonproteobacteria	DC28	Arcobacter nitrofigilis	95	6	M, R	Uncultured Arcobacter sp. clone OO.P2.OT.28	coastal water	HQ821621
Gammaproteobacteria	DC29	Agarivorans albus	99, 100	3	R, S	Uncultured bacterium clone NF038	marine sediment	JX391657
	DC30	Amphritea atlantica	89, 90	3	F	No reference	-	
	DC31	Amphritea japonica	96	1	R	Uncultured gamma proteobacterium clone 4DP1-G20	coral tissue	EU780381

DC32	Kangiella spongicola	90	2	F	No reference	-	
DC33	Pseudoalteromonas lipolytica	99	1	R	Uncultured bacterium clone Ev21-5-32	ciliate cell	MH556225
DC34	Pseudoalteromonas tetraodonis	99	1	F	Uncultured bacterium clone SPCiL-110	marine sponge	KC861114
DC35	Vibrio brasiliensis	100	2	S	Uncultured Vibrio sp. clone KR2_B04	surface water	AM183691
DC36	Vibrio mediterranei	100	1	F	Uncultured bacterium clone SanDiego_a6509	coral tissue	KF799776
DC37	Vibrio ponticus	95	2	M, R	Uncultured bacterium clone CD02009F01	coral tissue	HM768599

[a] The letters F, M, R, and S refer to used media, Fish extract, Marine, 1/10 diluted R2A and Sponge extract medium. See Experimental procedure for explanation of the different media used.
[b] The information of strains in GenBank composed of 16S rRNA gene sequences sharing over 98% identity to isolates are used as references.
