# Supplementary material for: Triggering Growth via Growth Initiation Factors in Nature: A Putative Mechanism for in situ Cultivation of Previously Uncultivated Microorganisms
Source: Front Microbiol. 2021 May 4;12:537194. doi: 10.3389/fmicb.2021.537194 (PMC8129545; doi:10.3389/fmicb.2021.537194)
Supplement: Supplementary file 3 [file Data_Sheet_3.PDF]

Table S2. Phylogenetic affiliations of isolates with the SDP method on the basis of 16S rRNA gene sequences.

Taxonomic group	Strain #	Closest species among cultured stains	Similarity	Number of isolates	Medium[a]	Closest sequences among uncultured / environmental sample[b]	Environmental source	Accession # of reference
Bacteroidetes	SDP1	Aquimarina atlantica	99	1	M	No reference	-	-
	SDP2	Maribacter dokdonensis	99	1	S	Uncultured Maribacter sp. clone C146001051	seawater	JX854260
	SDP3	Mesoflavibacter sabulilitoris	100	6	M, R, S	Uncultured bacterium clone PS13	seawater	MG001976
	SDP4	Tenacibaculum amylolyticum	96	1	S	No reference	-	-
Alphaproteobacteria	SDP5	Loktanella maritima	99	4	M	Uncultured bacterium clone S1B1S_11-079	marine sediment	KY190932
	SDP6	Phaeobacter leonis	100	5	S	Uncultured bacterium clone 135-76	marine sediment	MF978509
	SDP7	Pseudovibrio denitrificans	100	9	F, M, R	Uncultured bacterium clone PHF_2C-B7_N14	hydrothermal field	KJ149188
	SDP8	Ruegeria atlantica	99, 100	9	F, M, S	Uncultured alpha proteobacterium clone 3.1	sea urchin	AM930440
	SDP9	Shimia marina	99, 100	6	F, R, S	Uncultured bacterium clone 0785N7_3_60_D04777	seawater	KJ811931
	SDP10	Sphingopyxis marina	99	3	F, S	Uncultured alpha proteobacterium clone 1HP1-C7	coral tissue	DQ781320
Gammaproteobacteria	SDP11	Algicola bacteriolytica	100	5	F, R	Uncultured gamma proteobacterium clone TKTMmva-B4	seawater	AB571544
	SDP12	Pseudoalteromonas rubra	100	7	M, R	Uncultured bacterium clone MCR08.H12	seawater	LC435075
	SDP13	Vibrio panuliri	100	3	R, S	Uncultured bacterium clone FW_clone2	hydrothermal fluids	KT731382

[a] The letters F, M, R, and S refer to used media, Fish extract, Marine, 1/10 diluted R2A and Sponge extract medium. See Experimental procedure for explanation of the different media used.
[b] The information of strains in GenBank composed of 16S rRNA gene sequences sharing over 98% identity to isolates are used as references.
